# A unified definition of clinical anthracycline resistance breast cancer

**DOI:** 10.1054/bjoc.1999.0958

**Published:** 2000-01-18

**Authors:** X Pivot, L Asmar, A U Buzdar, V Valero, G Hortobagyi

**Affiliations:** Centre Antoine Lacassagne, 33 avenue de Vallombrose, Nice cedex 2, 06189, France; Department of Breast Medical Oncology, University of Texas MD Anderson Cancer Center, Houston, TX, USA

**Keywords:** anthracycline resistance, breast cancer, clinical definition

## Abstract

The purpose of the study was to determine the response rates (RR) and duration to second- and third-line chemotherapy programmes in patients with anthracycline-resistant breast cancer, utilizing various definitions of anthracycline resistance. This was a retrospective analysis performed on 1335 patients with metastatic breast cancer who participated in consecutive clinical trials of first line, anthracycline-containing combination chemotherapy (ACCC) at the University of Texas MD Anderson Cancer Center between July 1973 and April 1980. Anthracycline-resistant groups were identified using definitions of anthracycline resistance found in the literature: progressive disease as best response to ACCC (Group 1, *n* = 56 patients); progressive disease while receiving ACCC after an intervening response to the drug (Group 2, *n* = 84); progressive disease within 6 months of last dose of ACCC (Group 3, *n* = 233); and progressive disease within 12 months of last dose of ACCC (Group 4, *n* = 272). Second- and third-line therapies administered to these patients included methotrexate, doxorubicin, mitoxantrone, bisantrene, vinblastine, vindesine, melphalan, mitomycin, cisplatin, etoposide and others, but not taxanes. The distribution of patients' characteristics was similar between the four groups, as was the use of second- and third-line regimens. Response rate (RR) to second-line chemotherapy were 5% and 7.7% for Group 1 and Group 2 respectively. In contrast, RR to second-line chemotherapy were 21.6% and 15% for Group 3 and 4. The differences in response rate between the combination of Groups 1 and 2 and Groups 3 or 4 were significant (*P* = 0.005 and *P* = 0.04 respectively). These results indicate that strictly defined anthracycline resistance as defined in Groups 1 and 2 is associated with resistance to many other cytotoxic drugs. The definitions used in Groups 3 and 4 include many patients with responsive tumours, and a more favourable prognosis. © 2000 Cancer Research Campaign


*British Journal of Cancer* (2000) **82**(3), 529–534
© 2000 Cancer Research Campaign
DOI: 10.1054/ bjoc.1999.0958, available online at http://www.idealibrary.com on IDEAL®

### A unified definition of clinical anthracycline resistance breast cancer

**X Pivot[1], L Asmar[2], AU Buzdar[2], V Valero[2] and G Hortobagyi[2]**

[1]Centre Antoine Lacassagne, 33 avenue de Vallombrose, 06189, Nice cedex 2, France; [2]Department of Breast Medical Oncology, University of Texas MD Anderson Cancer Center, Houston, TX, USA

**Summary** The purpose of the study was to determine the response rates (RR) and duration to second- and third-line chemotherapy programmes in patients with anthracycline-resistant breast cancer, utilizing various definitions of anthracycline resistance. This was a retrospective analysis performed on 1335 patients with metastatic breast cancer who participated in consecutive clinical trials of first line, anthracycline-containing combination chemotherapy (ACCC) at the University of Texas MD Anderson Cancer Center between July 1973 and April 1980. Anthracycline-resistant groups were identified using definitions of anthracycline resistance found in the literature: progressive disease as best response to ACCC (Group 1, $n = 56$ patients); progressive disease while receiving ACCC after an intervening response to the drug (Group 2, $n = 84$); progressive disease within 6 months of last dose of ACCC (Group 3, $n = 233$); and progressive disease within 12 months of last dose of ACCC (Group 4, $n = 272$). Second- and third-line therapies administered to these patients included methotrexate, doxorubicin, mitoxantrone, bisantrene, vinblastine, vindesine, melphalan, mitomycin, cisplatin, etoposide and others, but not taxanes. The distribution of patients' characteristics was similar between the four groups, as was the use of second- and third-line regimens. Response rate (RR) to second-line chemotherapy were 5% and 7.7% for Group 1 and Group 2 respectively. In contrast, RR to second-line chemotherapy were 21.6% and 15% for Group 3 and 4. The differences in response rate between the combination of Groups 1 and 2 and Groups 3 or 4 were significant ($P = 0.005$ and $P = 0.04$ respectively). These results indicate that strictly defined anthracycline resistance as defined in Groups 1 and 2 is associated with resistance to many other cytotoxic drugs. The definitions used in Groups 3 and 4 include many patients with responsive tumours, and a more favourable prognosis. © 2000 Cancer Research Campaign

**Keywords:** anthracycline resistance; breast cancer; clinical definition

It is commonly believed by oncologists that anthracycline-containing regimens are the most effective combinations for the management of patients with breast cancer. Anthracycline-containing regimens represent the treatment of choice for adjuvant or neoadjuvant chemotherapy (Hortobagyi et al, 1991, 1993, 1995), and produce the highest objective response rates in metastatic breast cancer (Henderson, 1991). Unfortunately, however, anthracycline-containing chemotherapy regimens are (or become) ineffective in many patients.

Resistance of human breast cancers to anthracyclines results from the acquisition or pre-existence of several drug resistance mechanisms, including: reduction of the fluidity of the cell membrane, increase in the effectiveness of DNA repair mechanisms, multidrug resistance with overexpression of the Gp170 membrane glycoprotein, or modification of topoisomerase II activity (De Vita, 1993). These mechanisms of drug resistance are common to most chemotherapy agents. For this reason the presence of anthracycline resistance might indicate that no cytotoxic drug will have satisfactory results.

Several new cytotoxic agents, however, have been demonstrated to have definite anti-tumour effects in anthracycline-resistant tumours. This exciting characteristic is shared by mitomycin, most of the vinca alkaloids, including vinorelbine, and the most recently developed family of drugs with marked anti-tumour activity against breast cancer, the taxoids. A rapid overview of the results of these new chemotherapy agents in patients with breast cancer supports the observation that these drugs are useful for the treatment of anthracycline-resistant tumours. Table 1 reports published definitions of anthracycline-resistant breast cancer.

The criteria used to define anthracycline resistance among these trials varied. Therefore, it is difficult to determine the efficacy of these drugs in anthracycline-resistant tumours and to compare the relative activity of these drugs. The most stringent definition of anthracycline resistance was absence of response to a first- or a second-line anthracycline-containing regimen, or relapse during anthracycline-containing adjuvant or neoadjuvant chemotherapy (primary resistance). Secondary resistance was defined as an initial response followed by progressive disease during treatment with first- or second-line anthracycline-containing regimen, (Ravdin et al, 1995; Valero et al, 1995; Vermorken et al, 1995). However, other published definitions included not only progressive disease during anthracycline-containing chemotherapy, but also cases in which disease recurrence was detected within 6 or 12 months or even later after completion of adjuvant, neoadjuvant, or first-line metastatic anthracycline-containing regimen (Creech et al, 1983; Yau et al, 1985; Walter et al, 1992; Holmes et al, 1993; Nabholtz et al, 1993; Seidman et al, 1993; Degardin et al, 1994; Munzone et al, 1994; Wilson et al, 1994; Jones et al, 1995).

The major objective of the study reported here was to propose a unified clinical definition of anthracycline resistance in breast cancer.

*Received 11 May 1999*
*Revised 1 September 1999*
*Accepted 14 September 1999*

Correspondence to: X Pivot

**Table 1** Studies evaluating efficacy of cytotoxic agents in anthracycline-resistant breast cancer

| Reference | Agent | Number of patients | Definition of anthracycline resistance |
|---|---|---|---|
| Valero | Docetaxel | 34 | PD during ACR |
| Ravdin | Docetaxel | 35 | PD during ACR |
| Vermorken | Paclitaxel | 36 | PD during ACR |
| Seidman | Paclitaxel | 49 | PD during ACR |
| Munzone | Paclitaxel | 50 | PD during or within 12 months after ACR |
| Holmes | Paclitaxel | 18 | PD during ACR |
| Wilson | Paclitaxel | 33 | PD during or within 16 months after ACR |
| Nabholtz | Paclitaxel | 96 | PD during or within 6 months after ACR |
| Jones | Vinorelbine | 115 | PD during or any time after ACR |
| Degardin | Vinorelbine | 100 | PD during or any time after ACR |
| Yau | Vindesin | 61 | PD during or any time after ACR |
| Yau | Vinblastine | 23 | PD during or any time after ACR |
| Walters | Mitolycin | 67 | PD during or any time after ACR |
| Creech | Mitolycin | 90 | PD during or any time after ACR |

ACR = anthracycline-containing regimen; PD = progressive disease.

**Table 2** Number of patients treated with second- or third-line chemotherapy according to various definitions of anthracycline resistance among 1335 patients with metastatic breast cancer treated with first-line ACR

| Definition of anthracycline resistance | Number of patients | Number of patients treated with Second-line chemotherapy | Number of patients treated with Third-line chemotherapy |
|---|---|---|---|
| Primary resistance (PD during ACR with no intervening response) | 56 | 40 | 14 |
| Secondary resistance (PD during ACR with intervening response) | 84 | 65 | 26 |
| Primary + secondary resistance | 140 | 105 | 40 |
| PD within 6 months after last dose of ACR | 233 | 102 | 49 |
| PD between 6 and 12 months after last dose of ACR | 272 | 126 | 50 |

ACR: anthracycline-containing regimen; PD: progressive disease.

### PATIENTS AND METHODS

This study included 1335 patients treated at the University of Texas MD Anderson Cancer Center between July 1973 and April 1980 with an anthracycline-containing regimen as first-line chemotherapy for metastatic breast cancer. A complete description of this patient population has previously been reported (Hortobagyi et al, 1983).

Second- and third-line chemotherapy regimens included cytotoxic agents such as doxorubicin, methotrexate, vincristine, vinblastine, vindesine, mitoxantrone, bisantrene, melphalan, mitomycin C, cisplatin, etoposide, teniposide, peptichimio, pentostatin, anguidine, AMSA, 5-fluorouracil, L-asparginase and other less effective agents. At the time these patients were treated drugs such as vinorelbine, paclitaxel and docetaxel had not reached clinical trials. Within the study population, we defined four subgroups of patients with anthracycline-resistant disease according to the various definitions of anthracycline-refractory breast cancer found in the literature.

### Definitions of anthracycline resistance

The most stringent definition of anthracycline resistance in the literature was absence of response to a first- or a second-line anthracycline-containing regimen (disease progression, or stable disease followed by disease progression), or relapse while adjuvant or neoadjuvant anthracycline-containing chemotherapy. This type of resistance was referred to as 'primary anthracycline resistance'.

Secondary resistance was defined as initial response followed by progressive disease while receiving first- or second-line anthracycline-containing chemotherapy.

In other published reports, less stringent criteria of anthracycline resistance were used. Some reports included not only patients with no response or progressive disease during anthracycline-containing treatment but also patients who had progressive disease within 6 or even 12 months after completion of neoadjuvant, or adjuvant therapy, or first-line anthracycline-containing chemotherapy for metastatic disease.

### Definition of objective response

A complete response included the disappearance of all measurable and assessable disease, with no new lesion. Partial response was applied to patients with a decrease greater than or equal to 50% of measurable lesions with no progression of assessable disease and no new lesion. Responders combined patients who achieved complete response or partial response.

### Characteristics of patient subgroups

Of the 1335 patients in this study, 74 (5.5%) died during administration of the anthracycline-containing regimen. These patients were excluded from the analysis. The median follow-up for the study population was 27.5 months (range 3–255 months).

© 2000 Cancer Research Campaign

**Table 3** Patient characteristics and survival experience of patients with anthracycline-resistant breast cancer according to different definitions of anthracycline-resistance

| | | | | Definition of anthracycline resistance | |
|---|---|---|---|---|---|
| **Patient characteristics** | **Primary** | **Secondary** | **Primary+ secondary** | **Progression/relapse within 6 months after last dose of anthracycline therapy** | **Progression/relapse 6 to 12 months after last dose of anthracycline therapy** |
| Number of patients | 56 | 84 | 140 | 233 | 272 |
| Stage IV at presentation | 8 (14%) | 16 (19%) | 24 (17%) | 35 (15%) | 48 (17.6%) |
| Madian age at diagnosis (range) | 51 (24–74) | 48 (24–77) | 49 (24–77) | 49 (25–79) | 49 (25–78) |
| Madian age at recurrence (range) | 53 (25–75) | 53 (24–79) | 53 (24–79) | 52 (26–79) | 53 (25–79) |
| Median DFI in months (range) | 18 (1–144) | 27,5 (2–191) | 23 (1–191) | 17,5 (1–360) | 24 (1–275) |
| Median OS from diagnosis in months (range) | 28 (6–365) | 46 (7–231) | 39 (6–365) | 35 (7–410) | 42 (12–397) |
| Median OS from recurrence in months (range) | 14 (3–273) | 20 (6–141) | 18 (3–273) | 19 (7–235) | 25 (12–259) |
| Metastatic site | | | | | |
| CNS | 0 | 0 | 0 | 4 (1.7%) | 2 (0.7%) |
| Bone | 20 (35.7%) | 39 (46.4%) | 59 (42.1%) | 74 (31.8%) | 126 (46.3%) |
| Lung | 13 (23.2%) | 37 (44%) | 50 (35.7%) | 53 (22.7%) | 82 (30.1%) |
| Liver | 7 (12.5%) | 14 (16.6%) | 21 (15%) | 24 (10.3%) | 38 (14%) |
| Soft-tissues | 20 (35.7%) | 42 (50%) | 62 (44.3%) | 116 (49.8%) | 110 (40.5%) |

CNS: central nervous system; DFI: disease-free interval; OS: overall survival.

Fifty-six of the 1335 patients in the study (4.2%) had primary anthracycline resistance, and 84 patients (6.3%) had secondary anthracycline resistance. An additional 233 patients (17.5%) had progressive disease within 6 months of completion of an anthracycline-containing regimen, and an additional 272 patients (20.4%) had disease progression between 6 and 12 months after the last dose of an anthracycline-containing regimen. The number of patients in each of these subgroups who received second- or third-line chemotherapy is given in Table 2.

Other characteristics of each subgroup are given in Table 3. The patients included in this analysis received no adjuvant or neoadjuvant treatment, and the distribution of first-line chemotherapy programmes and number and type of systemic treatments that patients received after first-line anthracycline-containing regimens were similar in each subgroup.

### Statistical analysis

For each of the four subgroups, the response rate (RR) to second- and third-line chemotherapy regimens were available. A comparison between the four subgroups was performed using the $\chi^2$ test. The RR for each group were also combined with the aim of describing a population similar to the one selected by previous authors.

The overall survival was measured from the date of progression on anthracycline-containing therapy until death from any cause, or until the date of last follow-up for patients still alive. The length of progression-free survival was defined at the time from initiation of chemotherapy to the time of documented disease progression. Curves plotting the distribution of disease-free and overall survival times were calculated by the method of Kaplan and Meier (1958), and differences among distributions were tested using the log-rank test (Mantel, 1966). *P*-values of 0.05 or less were considered statistically significant and strong statistical evidence against the null hypothesis.

### RESULTS

There were no differences in the distribution of patients' characteristics between the four subgroups analysed. The types of first-line anthracycline-containing regimens, and the number and type of salvage treatment used after first-line chemotherapy were similar in all four groups.

Table 4 reports the RR by line of chemotherapy for each subgroups. In the subgroup of patients with stringently defined primary or secondary anthracycline-resistant breast cancer, the RR to second- and third-line chemotherapy were 6.7% (7/105), and 7.5% (3/40) respectively.

Among patients who had recurrent disease within 6 months after completion of a first-line anthracycline-containing regimen (excluding patients with primary or secondary anthracycline-resistant breast cancer), the RR after second- and third-line chemotherapy were 21.6% (22/102), and 14.3% (7/49) respectively. The differences in RR between this third group and the combination of the two prior groups were statistically significant for second-line chemotherapy ($P = 0.005$), but not significant for third-line chemotherapy ($P = 0.3$).

Among patients with a relapse that occurred between 6 and 12 months after completion of first-line chemotherapy. The RR reported for second- and third-line chemotherapy were 15% (19/126), and 12% (6/50) respectively. Again, the differences in RR between this group and the combination of the first two groups (primary and secondary anthracycline-resistance) were statistically significant ($P = 0.04$) for second-line chemotherapy, but not for third-line chemotherapy ($P = 0.5$).

The RR achieved by second- and third-line chemotherapy in the subgroup of patients who recurred within 6 months and between 6 and 12 months after completion of first-line chemotherapy were not statistically different ($P = 0.2$ and $P = 0.8$ respectively). To select a population comparable to those utilized in other published

*© 2000 Cancer Research Campaign*

**Table 4** Response rates for second- and third-line chemotherapy according to definition of anthracycline resistance

| Definition of anthracycline resistance | Objective responses (%) for | | | |
|---|---|---|---|---|
| | Second-line chemotherapy | | Third-line chemotherapy | |
| Primary resistance (PD during ACR with no intervening response) | 5% (Cl: 6.7%) | (2/40) | 7.1% (Cl: 13.5%) | (1/14) |
| Secondary resistance (PD during ACR with intervening response) | 7.7% (Cl: 6.5%) | (5/65) | 7.7% (Cl: 10%) | (2/26) |
| Primary + secondary resistance | 6.7% (Cl: 4.8%) | (7/105) | 7.5% (Cl: 8.2%) | (3/40) |
| PD within 6 months after last dose of ACR | 21.6% (Cl: 8%) | (22/102) | 14.3% (Cl: 9.8%) | (7/49) |
| PD between 6 and 12 months after last dose of ACR | 15% (Cl: 6.2%) | (19/126) | 12% (Cl: 9%) | (6/50) |
| No anthracycline resistance | 21.7% (Cl: 4.4%) | (75/345) | 14.3% (Cl: 5.8%) | (21/144) |

ACR: anthracycline-containing regimen; PD: progressive disease.

**Table 5** Survival of patients with anthracycline-resistant breast cancer according to the various definitions of resistance

| Anthracycline-resistant subgroup | Median survival (range) | Survival (s.e.) (%) | | |
|---|---|---|---|---|
| | | 1-year | 2-year | 3-year |
| Primary resistance + secondary resistance subgroup | 5 months (1–78) | 21% (4%) | 9% (4%) | 1% (2%) |
| PD within 6 months after last dose of ACR | 9 months (0–198) | 35% (3%) | 13% (3%) | 6% (2%) |
| PD between 6 to 12 months after last dose of ACR | 11 months (1–201) | 39% (3%) | 14% (3%) | 7% (2%) |

PD: progressive disease; s.e.: standard error; ACR: anthracycline-containing regimen.

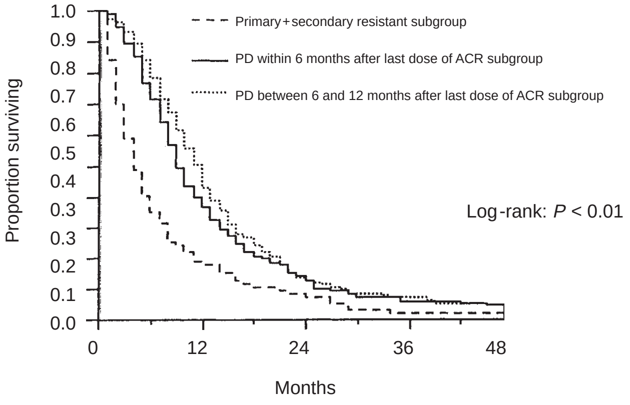

**Figure 1** Survival from progression on an anthracycline-containing regimen until death or date of last follow-up studies of anthracycline-resistant breast cancer, we have to combine the results of the first three subgroups or the results of all four subgroups (Table 4). It is evident that the inclusion of patients who developed progressive disease up to 6 or 12 months after the last dose of anthracycline considerably improves response rates to second- and third-line therapies.

There was a statistically significant difference in survival ($P < 0.01$) from the date of progression among the three subgroups (Figure 1). Survival at 1, 2 and 3 years from the date of progression on (or after) anthracycline-containing therapy for the four groups is given on Table 5.

### DISCUSSION

According to the definitions of anthracycline resistance in the literature, we selected four subgroups for analysis from our database of 1335 patients treated in prospective clinical trials of anthracycline-containing first-line chemotherapy for metastatic breast cancer. No differences were noted in terms of patient and treatment characteristics between these four subgroups and the total group of 1335 (Hortobagyi et al, 1983).

For the groups of patients whose relapses occurred within 6 months or between 6 and 12 months after completion of an anthracycline-containing regimen, the RR to second- (21.5% and 15%) and third-line (14.3% and 12%) chemotherapy were similar between the two subgroups and consistent with the results in the literature for patients previously treated (but not necessarily resistant to) with chemotherapy. In the literature, RR reported for second- or third-line chemotherapy for metastatic breast cancer are between 17% and 54% (Hortobagyi et al, 1995), depending on the type of chemotherapy used; the mean RR for second- or third-line chemotherapy without an anthracycline is 20%. Moreover, the RR to second- (21.5% and 15%) and third-line chemotherapy (14.3% and 12%) found in these subgroups with loosely defined anthracycline resistance, were not different from the rate reported for the rest of the total group of 1335 patients (21.7% and 14.4% respectively, for second- and third-line chemotherapy).

The anthracycline-resistant phenotype requires several non-specific mechanisms of resistance. As a consequence, the lack of efficacy of an anthracycline-containing regimen predicts a reduction in the efficacy for most other chemotherapy drugs. Disease relapse within 12 months after the completion of anthracycline

*© 2000 Cancer Research Campaign*

treatment may represent partial resistance to anthracyclines and other agents, or may indicate kinetic characteristics of the disease that would result in accelerated regrowth after effective chemotherapy. However, patients with disease relapse within 12 months after anthracycline-containing chemotherapy do not represent a specific population with strong chemotherapy-resistant characteristics. This finding is also consistent with the fact that the RR in these two groups were not different from those expected or obtained in the overall population studied. It seems reasonable not to consider these two patient subgroups as having anthracycline-resistant breast cancer.

Recurrence or metastases that occurred shortly (within months) after completion of therapy, are probably a sign of reduced efficacy of the anthracycline-containing regimen, or an indication of a particularly aggressive and rapidly growing tumour. These characteristics do not preclude sensitivity to anthracyclines, and does not represent proof of anthracycline-resistance.

The patients defined as having primary and secondary anthracycline resistance exhibited significantly lower rates of RR after second-line (6.7%) or after third-line chemotherapy (7.5%). These RR were statistically lower for the second-line chemotherapy than those obtained in the rest of the population after similar salvage therapies. The differences in RR at third-line chemotherapy were not found to be statistically significant, probably related to smaller sample size inducing lower power and less precise estimates. Therefore, it seems that the definition of anthracycline resistance that includes only progressive disease during treatment with an anthracycline selects a very unfavourable group, that is clearly less responsive to other chemotherapy regimens and reflects true anthracycline resistance.

This study included only patients with metastatic breast cancer treated with an anthracycline-containing regimen; however, the criteria for same anthracycline resistance could probably be extended to patients receiving anthracycline-containing adjuvant and neoadjuvant treatment. In these cases, our definition would indicate that relapse that occurred within 6 or 12 months of the last dose of anthracycline therapy is not a marker of anthracycline resistance, and only progressive disease during a neoadjuvant or adjuvant anthracycline-containing regimen is an acceptable indicator to identify anthracycline resistance.

These results apply to cytotoxic agents available during the study period. Response to newer, and possibly more effective, agents, such as taxanes (Pivot et al, 1999) will need to be examined prospectively, preferably with a clear definition of anthracycline-resistant populations treated.

### CONCLUSION

A clinical definition of anthracycline-refractory breast cancer that includes only patients with progressive disease during anthracycline chemotherapy seems to determine a very unfavourable subset, that is significantly less sensitive to other chemotherapy agents. All other definitions with broader inclusion criteria appear to determine populations that will have response rates similar to those achieved by the total population with metastatic breast cancer. To determine the real activity of cytotoxic drugs in anthracycline-resistant breast cancer, all previously reported studies that have analysed drug activity against anthracycline-resistant breast cancer may need to be re-evaluated.

### ACKNOWLEDGEMENTS

Supported in part by Nylene Eckles professorship in Breast Cancer Research. The authors thank Stephanie Deming for editorial assistance, and Debbie Geller for typing and formatting the manuscript.

### REFERENCES

Creech R, Catalano R, Shah M and Dayal H (1993) An effective low-dose mitomycin regimen for hormonal- and chemotherapy-refractory patients with metastatic breast cancer. *Cancer* **51**: 1034–1040

Degardin M, Bonneterre J, Hecquet B, Pion JM, Adenis A, Horner D and Demaille A (1994) Vinorelbine (Navelbine) as a salvage treatment for advanced breast cancer. *Ann Oncol* **5**: 423–426

De Vita V (1993) Biochemical resistance to chemotherapy is the major impediment to successful treatment. In: *Cancer: Principles and Practice of Oncology*, De Vita V, Hellman S and Rosenberg S (eds), pp. 281–283. Lippincott: Philadelphia

Henderson C (1991) Chemotherapy for metastatic disease. In: *Breast Disease*, Harris J, Hellman S, Henderson C and Kinne D (eds), pp. 604–665. Lippincott: Philadelphia

Holmes A, Valero V, Walters R, Theriault R, Booser D, Fraschini G, Buzdar A, Frye D, Gibbs H and Hortobagyi G (1993) The MD Anderson Cancer Center experience with Taxol in metastatic breast cancer. *Cancer* **15**: 161–169

Hortobagyi G (1995*a*) Management of breast cancer: status and future trends. *Semin Oncol* **22**: 101–107

Hortobagyi G (1995*b*) Future directions for vinorelbine (Navelbine). [Review]. *Semin Oncol* **22**: suppl 2: 80–86

Hortobagyi G and Buzdar A (1991) Locally advanced breast cancer: a review including the MD Anderson experience. In: *High-risk Breast Cancer*, Ragaz J and Ariel I (eds) pp. 382–415. Springer-Verlag: Berlin

Hortobagyi G and Buzdar A (1993) Present status of anthracyclines in adjuvant treatment of breast cancer [Review]. *Drugs* 45, suppl **2**: 10–19

Hortobagyi G and Buzdar A (1995) Current status of adjuvant systemic therapy for primary breast cancer: progress and controversy. *CA Cancer* **45**: 199–226

Hortobagyi G, Smith T, Legha S, Swenerton K, Gehan E, Yap H, Buzdar A and Blumenschein G (1983) Multivariate analysis of prognostic factors in metastatic breast cancer. *J Clin Oncol* **12**: 776–786

Jones S, Winner E, Vogel C, Laufman L, Hutchins L, O'Rourke M, Lembersky B, Budman D, Bigley J and Hohneker J (1995) Randomized comparison of vinorelbine and melphalan in anthracycline-refractory advanced breast cancer. *J Clin Oncol* **13**: 2567–2574

Kaplan E and Meier P (1958) Non-parametric estimation from incomplete observations. *J Am Stat Assoc* **53**: 457–481

Mantel N (1966) Evaluation of survival data and two new rank order statistics arising in its consideration. *Cancer Chemother Rep* **50**: 163–170

Munzone E, Capri G, Fulfaro F, Tarenzi E, Caraceni A, Villani F, Spreafico C, Laffranchi A, Crabeels D and Gianni L (1994) Paclitaxel by 3 H schedule in relapsed breast cancer resistant to anthracycline. *Ann Oncol* **5**: 43 (abstract 212).

Nabholtz J, Gelmon K, Bontenal M, Spielmann M, Clavel M, Seeber S, Conte P, Boneterre J, Fumoleua P, Slukes A, Sauter C, Roche H, Calvert H, Kaufman J, Chazard M, Diergarten K, Gallant G, Thompson M, Winograd B, Onetto N and Bristol-Myers Squibb Taxol Study Group (1993) Randomized trial of two doses of taxol in metastatic breast cancer: an interim analysis. *Proc Am Soc Clin Oncol* **12**: 60 (abstract 42)

Pivot X, Asmar L and Hortobagyi G (1999) The efficacy of chemotherapy with docetaxel and paclitaxel in anthracycline-resistant breast cancer. *Int J Oncol* **15**: 381–386

Radvin P, Burris H, Cook G, Eisenberg P, Kane M, Bierman W, Mortimer J, Genevois E and Gellet R (1995) Phase II trial of docetaxel in advanced anthracycline-resistant or anthracenedione-resistant breast cancer. *J Clin Oncol* **13**: 2879–2885

Seidman A, Norton L, Reichman B, Crown J, Yao T, Heelan R, Hakes T, Lebwohl D, Gilewski T and Surbone A (1993) Preliminary experience with paclitaxel plus recombinant human granulocyte colony-stimulating factor in the treatment of breast cancer. *Semin Oncol* **20**: 40–45

Valero V, Holmes F, Waters R, Theriault R, Esparza L, Fraschini G, Fonseca G, Bellet R, Buzdar A and Hortobagyi G (1995) Phase II trial of docetaxel: a new, highly effective antineoplasic agent in the management of patients with anthracycline-resistant metastatic breast cancer. *J Clin Oncol* **13**: 2886–2894

Vermoken JB, Ten Bokkel Huinink W, Mandjes I, Postman T, Huizing T, Heimans J, Beijnen J, Bierhorst F, Winograd B and Pinedo H (1995) High dose paclitaxel with granulocyte colony-stimulating factor in patients with advanced breast

*© 2000 Cancer Research Campaign* 

carcinoma refractory to anthracycline therapy: a European Cancer Center trial. *Semin Oncol* **22**: 16–22
Walters R, Frye D, Buzdar A, Holmes F and Hortobagyi G (1992) A randomized trial of two dosage schedules of mitomycin C in advanced breast carcinoma. *Cancer* **69**: 476–481
Wilson W, Berg S, Bryant G, Wittes R, Bates S, Fojo A, Steinberg S, Goldspiel B, Herdt J, O'Shaughnessy J, Balis F and Chabner B (1994) Paclitaxel in doxorubicin-refractory or mitaxantrone refractory breast cancer: a phase I/II trial of 96-hour infusion. *J Clin Oncol* **12**: 1621–1629
Yau J, Yap Y, Buzdar A, Hortobagyi G, Bodey G and Blumenschein G (1985) A comparative randomized trial of vinca-alcaloïds in patients with metastatic breast carcinoma. *Cancer* **55**: 337–340

 *© 2000 Cancer Research Campaign*
